# Dual physiological responsive structural color hydrogel particles for wound repair

**DOI:** 10.1016/j.bioactmat.2025.01.002

**Published:** 2025-01-07

**Authors:** Li Wang, Lu Fan, Anne M. Filppula, Yu Wang, Feika Bian, Luoran Shang, Hongbo Zhang

**Affiliations:** a Joint Centre of Translational Medicine, Wenzhou Key Laboratory of Interdiscipline and Translational Medicine, The First Affiliated Hospital of Wenzhou Medical University, Wenzhou, China; b Shanghai Xuhui Central Hospital, Zhongshan-Xuhui Hospital, and the Shanghai Key Laboratory of Medical Epigenetics, the International Co-laboratory of Medical Epigenetics and Metabolism (Ministry of Science and Technology), Institutes of Biomedical Sciences, Fudan University, Shanghai, 200032, China; c Pharmaceutical Sciences Laboratory, Åbo Akademi University, Turku, 20520, Finland; d Turku Bioscience Centre, University of Turku and Åbo Akademi University, Turku, 20520, Finland; e Wenzhou Institute, University of Chinese Academy of Sciences, Wenzhou, Zhejiang, 200032, China

**Keywords:** Dual responsiveness, Structural color, Inverse opal, Wound repair, Microparticles

## Abstract

Hydrogel-based patches have demonstrated their values in diabetic wounds repair, particularly those intelligent dressings with continuous repair promoting and monitoring capabilities. Here, we propose a type of dual physiological responsive structural color particles for wound repair. The particles are composed of a hyaluronic acid methacryloyl (HAMA)-sodium alginate (Alg) inverse opal scaffold, filled with oxidized dextran (ODex)/quaternized chitosan (QCS) hydrogel. The photo-polymerized HAMA and ionically cross-linked Ca-Alg constitute to the dual-network hydrogel with stable structural color. Furthermore, the ODex/QCS hydrogel, combined with glucose oxidase (GOX), exhibits pH/glucose dual responsiveness. Moreover, antimmicrobial peptide (AMP) plus vascular endothelial growth factor (VEGF) are comprised within the GOX-doped ODex/QCS hydrogel. In the high-glucose wound environment, GOX catalyzes glucose to generate acidic products, triggering rapid release of AMP and VEGF. Importantly, this process also leads to structural color changes of the particles, offering significant potential for wound monitoring. It has been demonstrated that such particles greatly promote the healing progress of diabetic wound *in vivo*. These results indicate that the present dual responsive particles would find valuable applications in diabetic wounds repair and the associated areas.

## Highlights

• Stable structural color scaffold with improved mechanical property are presented.
• GOX-doped ODex/QCS hydrogel exhibit dual physiological responsive.
• Structural color property of the particles offers significant potential for wound monitoring.
• The presented particles greatly promote the healing progress of diabetic wound *in vivo*.

## Graphical abstract

We fabricated the hydrogel particle system comprising a hyaluronic acid methacrylate (HAMA)-sodium alginate (Alg) inverse opal scaffold, refilled with dynamic hydrogel for diabetic wound healing. Furthermore, this dynamic hydrogel exhibits pH/glucose dual responsiveness. Meanwhile, the drug release behavior could be monitored by structural color property. It has been demonstrated that such particles greatly promote the healing progress of diabetic wound *in vivo*.

**Figure:**
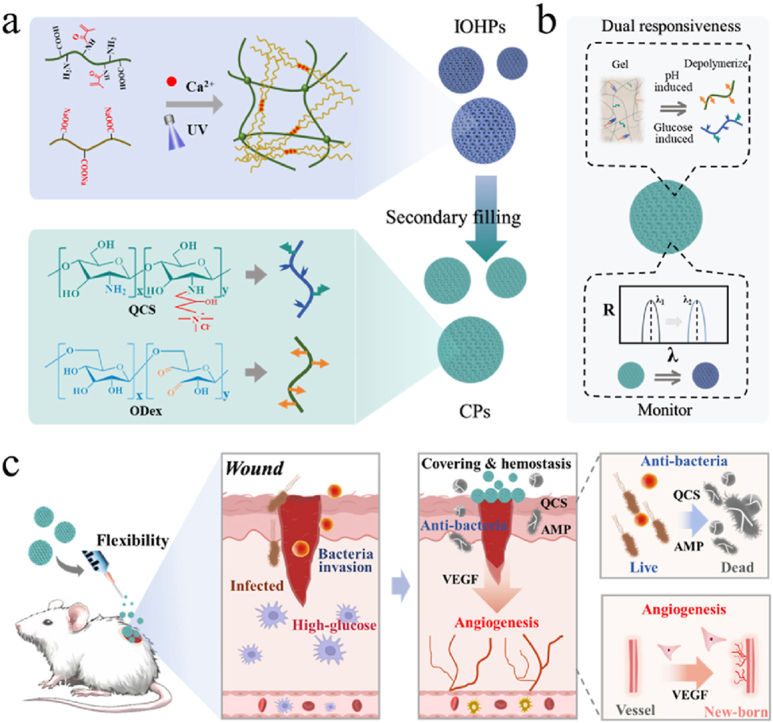

## 1 Introduction

Diabetic wounds have emerged as one of the most challenging health issues due to their complex pathophysiology [[1], [2], [3], [4]]. Various active substances have been developed to solve these problems, such as antibacterial agents like antimicrobial peptide (AMP) [5,6]. Additionally, drugs that promote angiogenesis, for instance vascular endothelial growth factor (VEGF) [7,8], are often used with antimicrobial agents for combinatory treatment [9,10]. To effectively deliver these drugs, numerous systems have been investigated, especially hydrogel-based wound dressings [[11], [12], [13], [14], [15], [16]]. Moreover, it has been demonstrated that hydrogel dressings with responsiveness to the external environment can achieve dynamic drug release and better solve the practical challenges [[17], [18], [19]]. Despite much process, hydrogel dressings with responsiveness to single stimuli are insufficient to coordinate with the complex physiological environment of diabetic wounds [[20], [21], [22]]. Besides, due to the limitation of the structural design, traditional bulk hydrogels often cover the entire wound, which may hamper the oxygen permeation and is thus not conducive to wound repair [[23], [24], [25]]. Thus, there is a considerable demand for hydrogel dressings that possess effective responsiveness and monitoring abilities, as well as intricate structural design.

**Fig. 1:**
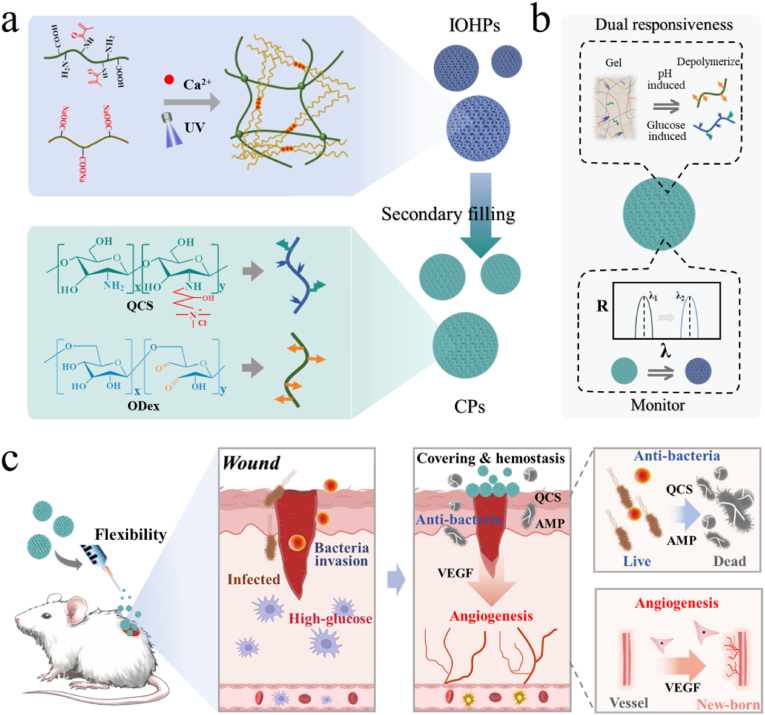
Schematic of CPs preparation and their application in wound healing. a-b) Chemical structure of hydrogel network of inverse opal and secondary infusion material. c) Composite particles applied in wound management. From left to right: Bacterial infected wound, CPs covering and inhibiting bacteria, and CPs promoting wound healing.

In this paper, we proposed a dual physiological responsive inverse opal particle with intelligent responsiveness behavior and an ideal monitoring function for wound repair, as illustrated in Fig. 1. Inverse opal, characterized with an ordered interconnected porous framework, exhibits large specific surface area and customizable structural color properties [[26], [27], [28], [29]]. Their special structure allows them to serve as a smart self-reporting platform as well as delivery vehicles for various active components. However, the simple inverse opal scaffold is insufficient to respond promptly to the complex microenvironment of diabetic wounds [30]. To improve the responsiveness, an effective approach is the use of a secondary filling material. Dynamic hydrogel based on Schiff base bond[[31], [32], [33], [34]], in conjunction with highly catalytic glucose oxidase (GOX) [35], exhibits excellent pH/glucose dual responsiveness behavior, which can respond to high-glucose environment and the pH change caused by bacterial infection. Therefore, combining the inverse opal scaffold with the dual responsive dynamic hydrogel holds huge promise for developing a novel composite particle system with monitoring abilities for wound repair.

Herein, we fabricated the hydrogel particle system comprising a hyaluronic acid methacrylate (HAMA)-sodium alginate (Alg) inverse opal scaffold, refilled with dynamic hydrogel for diabetic wound healing. Within this system, the photo-polymerized HAMA and ionically cross-linked Alg constituted the dual-network hydrogel, which provided the inverse opal scaffold with enhanced mechanical stability. Additionally, the dynamic hydrogel, comprised of oxidized dextran (ODex), quaternized chitosan (QCS), and GOX, endowed the inverse opal particles with pH/glucose dual responsiveness. Moreover, AMP and VEGF were encapsulated in the GOX-doped ODex/QCS hydrogel. When applied in wound, such particle system reacts to the high-glucose environment of the wound. GOX efficiently catalyzes glucose to produce acidic gluconic acid, which then triggers the rapid switching of Schiff base bonds. This process causes the swift release of the encapsulated drugs. Importantly, this process also leads to changes in the particle's structural color because of the change of the refractive index, offering significant potential for delivery monitoring. Based on these unique features, our proposed particle system has been demonstrated to serve as an intelligent responsive dressing for wound management and monitoring. These results underscore the worthy applications of such particle systems in the biomedical field.

## 2 Results and discussion

**Fig. 2:**
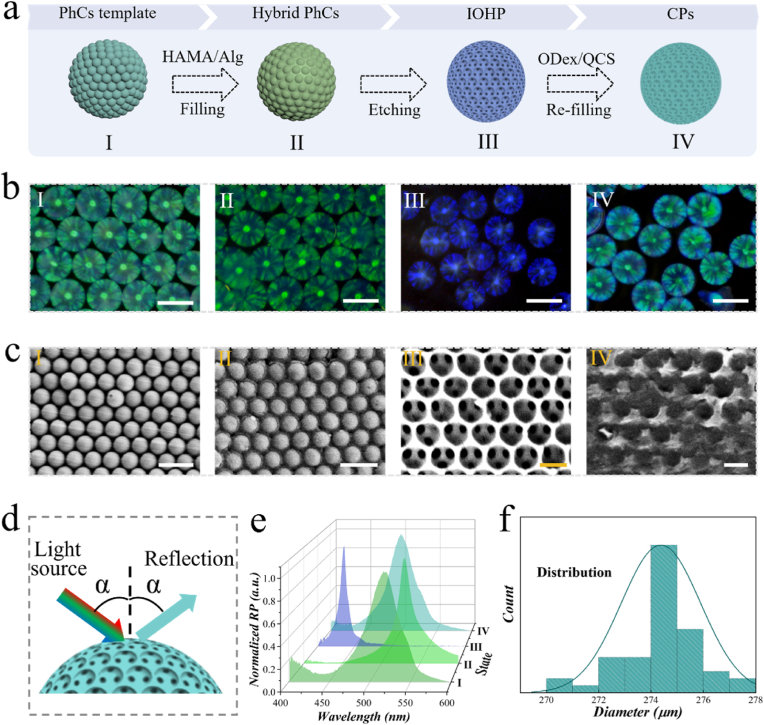
a) Scheme of the preparation of the dual responsive CPs. b) Microscopic images of the corresponding particles during the fabrication route. Scale bar is 300 μm. c) SEM pictures of the microstructure in corresponding particles. Scale bars are 500 nm in I and II, and 250 nm in III and IV. d) Schematic diagram of the reflection light of structural color. e) Reflection spectra of different particles corresponding to each stage. f) Diameter distribution of CPs.

The dual-responsive structural color hydrogel particles were fabricated according to the scheme shown in Fig. 2a. Firstly, silica nanoparticles self-assembled into photonic crystal (PhC) particles through droplet microfluidic technology. Then, photocurable HAMA and ionic-crosslinkable Alg were chosen as the main component to reversely replicate the structure of the PhC. To obtain inverse opal hydrogel particles (IOHPs), silica nanoparticles were etched by hydrofluoric acid. The dried IOHPs were fully immersed in the mixed solution of oxidized dextran (ODex), glucose oxidase (GOX) and quaternized chitosan (QCS). When solution components completely polymerized, the dynamic hydrogel was filled in the voids of IOHP, thus forming the composite particles (CPs). Compared with IOHP, the particle size of CPs was slightly larger (Fig. S1). The microscope images of different particles involved in the fabrication process were recorded and shown in Fig. 2b. To explore the micro-morphology, scanning electron microscope (SEM) was used to study particles. As indicated in Fig. 2c, silicon dioxide nanoparticles were distinctly observed to be arranged in a hexangular close-packed structure. The interstices between the nanoparticles can be thoroughly infiltrated by hydrogel. Following the removal of silica, the IOHP also maintained its ordered nanostructure, allowing another hydrogel material to fill the space formerly occupied by silica.

Due to the organized nanostructure, PhC exhibited characteristic photonic bandgaps, thereby displaying structural color. For the IOHP and CPs, they fundamentally retained the characteristic of a periodically alternating refractive index structure. Thus, both IOHP and CPs inherited the structural color features. Fig. 2d illustrated the reflection pattern of the structural color particles when illuminated by a light source covering the entire wavelength range. It is noteworthy that this reflection pattern can not only be observed by naked eyes, but also measured by a precise spectrometer. The reflection characteristic peaks of these particles were documented in Fig. 2e. This detectability held significant potential for subsequent monitoring. Moreover, benefiting from the stability of the microfluidic technology, the fabricated PhC particles exhibited a high degree of sphericity and uniform size. Consequently, the derived CPs inherited these characteristics. In a typical experiment, the diameter of the fabricated CPs was primarily concentrated around 274 μm (Fig. 2f). This feature ensured the batch stability of the particles, which was beneficial for further applications.

**Fig. 3:**
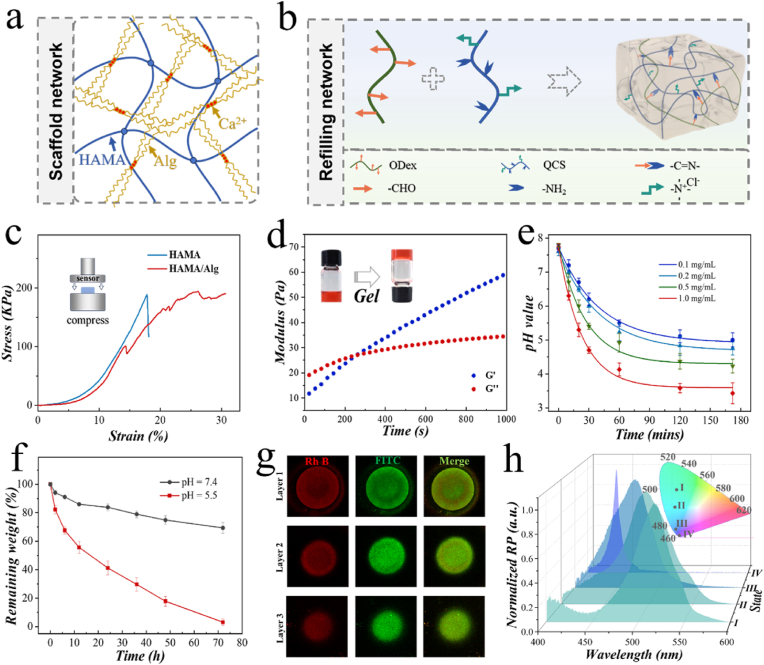
a) Illustration of the dual network structure of the inverse opal scaffold. b) Illustration of the Schiff base linkage formed by ODex and QCS. c) Compressive strain-stress curve of HAMA and HAMA/Alg. d) Time-dependent modulus analysis of the hydrogel formed by ODex and QCS upon mixing. e) Change of pH value of glucose solution with the presence of GOX-doped ODex/QCS hydrogel containing different concentrations of GOX. f) Analysis of remaining weight of GOX-doped ODex/QCS hydrogel in PBS buffer with different pH value. g) CLSM images of a particle encapsulating FITC-BSA and Rh B-labeled AMP. h) Corresponding reflection spectrum of CPs during CPs immersing in PBS (pH 5.5). I, II, III, and IV represented the distinct stages of CPs during the degradation process of ODex/QCS hydrogels.

The inverse opal scaffolds utilized a double network design, as shown in Fig. 3a. HAMA is a classic photo-polymerizable polymer. By introducing Alg polymer into the HAMA network, the mechanical property was optimized. The pre-gel solution containing HAMA and Alg was fully filled into the PhC template. The HAMA polymer was first polymerized using a UV source. Then, the entire system was immersed in a calcium chloride solution, allowing Alg to fully chelate with calcium ions and form a gel. To further investigate the impact of Alg, we conducted compression tests on single-component and double-network hydrogels. As shown in Fig. 3c, the single-network hydrogel exhibited a sharp drop in stress at 17.7 % strain, indicating overall fracture. In contrast, the double-network hydrogel prevented overall fracture, probably by inhibiting the growth of microcracks. This result demonstrated the better toughness of the double-network structure.

To endow the system with intelligent responsiveness to complex physiological environments, the secondary infusion hydrogel was mainly composed of ODex and QCS (Fig. 3b). The aldehyde groups from ODex and the amino groups from QCS could form dynamic chemical bonds. Rheological test of ODex/QCS showed that the storage modulus gradually became greater than the loss modulus, indicating gel formation (Fig. 3d). The images of the formed hydrogel were also rescored in Fig. S2. Additionally, the ODex/QCS system remained in a liquid state and was unable to form a gel in a mildly acidic environment (Fig. S3). Moreover, since both GOX and peptide drugs contain amino groups (abbreviated as R-NH_2_), we investigated the effect of their addition on the ODex/QCS hydrogel. The chemical states of ODex/QCS and QDex/QCS&R-NH_2_ were explored across to x-ray photoelectron spectroscopy (XPS). Compared to the C 1s spectrum of ODex/QCS hydrogel, the proportion of C3 (C=N) peaks significantly increased in the QDex/QCS&R-NH_2_ spectra, suggesting the enhancement of the dynamic bonds (Fig. S4). Moreover, in the N 1s spectrum of QDex/QCS&R-NH_2_ hydrogel, the proportion of C=N peaks increased, while the peaks corresponding to quaternary ammonium nitrogen significantly decreased (Fig. S5). These results indicated that GOX and peptide drugs were successfully encapsulated in the hydrogel.

Except with pH responsiveness, the incorporation of GOX also endowed the ODex/QCS hydrogel with glucose responsiveness (Fig. S6). Utilizing the high catalytic activity of GOX, glucose can be consumed to produce gluconic acid. This process causes a decrease in pH, which triggers hydrolysis of the Schiff base linkages. We investigated the catalytic ability of the GOX-doped ODex/QCS hydrogel under different GOX concentrations. As shown in Fig. 3e, in the presence of glucose, the GOX-doped ODex/QCS hydrogel exhibited excellent catalytic activity, quickly lowering the pH value of the system to below 6. To explore the pH responsiveness of the GOX-doped ODex/QCS hydrogel, we tested its stability in slightly acidic and neutral PBS solutions. Upon immersion, the GOX-doped ODex/QCS hydrogel degraded to a large extent in a pH 5.5 environment in three days, while the same hydrogel remained stable in a pH 7.4 environment (Fig. 3f). Besides, the effect of glucose on the degradability of the GOX-doped ODex/QCS hydrogel was also investigated. As shown in Fig. S7, the degradation ratio significantly accelerated in the glucose solution, which can be attributed to the pH decrease. These characteristics made the hydrogel suitable for controlled drug release in response to pH and glucose.

For drug encapsulation and release experiments, FITC-BSA was used as a substitute for VEGF and Rhodamine B (Rh B) was used to graft AMP. Both agents were loaded in GOX-doped ODex/QCS hydrogel. According to the confocal laser scanning microscope (CLSM) images, the whole particle emitted fluorescence, indicating the uniform payload of the drugs (Fig. 3g). According to the *in vitro* drug release test results, it was obvious that a mildly acidic environment could promote the rapid drug release from the hydrogel. Under neutral conditions, the release rate was only around 20 %, whereas in a mildly acidic environment, it reached up to 80 % in 72h (Fig. S8). Moreover, the presence of glucose also promoted the rapid release of AMP, which is due to the highly efficient catalytic ability of GOX (Fig. S9). Notably, for the CPs, as the ODex/QCS hydrogel degraded from the CPs in PBS (pH 5.5), the structural color of the CPs and their corresponding reflection peaks also changed accordingly. The reflection peaks of the particles were recorded using a spectrometer, and the corresponding standard color chart positions were also marked in Fig. 3h. This property showed the great potential of the structural color CPs as a visual monitoring platform.

**Fig. 4:**
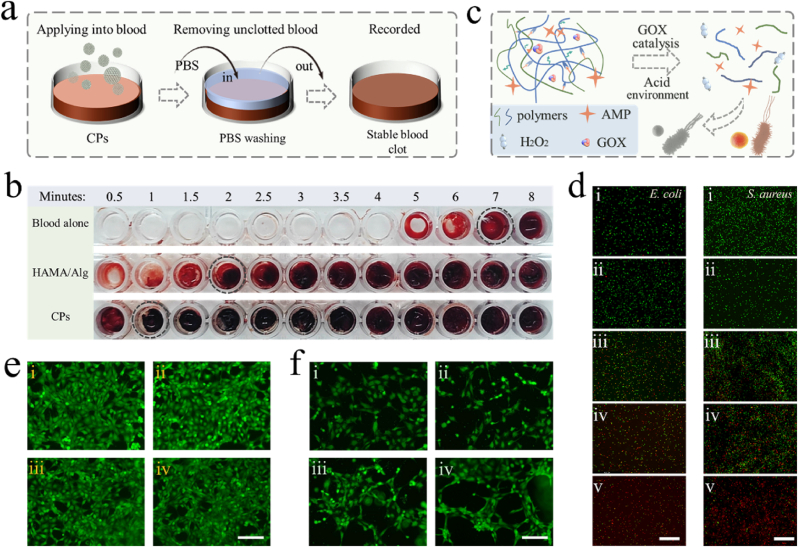
a) Schematic diagram of the *in vitro* coagulation test. b) Coagulation test results in different groups. c) Illustration of the dual-responsive release of AMP and the antibacterial property. d) Live/Dead florescent staining of *E. coli* and *S. aureus* suffered from different treatments. i: PBS group; ii: HAMA/Alg hydrogel group; iii: the pure CPs group (without GOX); iv: the AMP-loaded CPs group (without GOX); v: the AMP-loaded CPs group (with GOX). Scale bars are 100 μm. e) Florescent images of 3T3 cells in different groups. i: control group; ii: HAMA/Alg hydrogel group; iii: ODex/QCS hydrogel group; iv: HAMA/Alg and ODex/QCS composite material group. Scale bar is 50 μm. f) Florescent images of angiogenesis in different groups. i: control group; ii: blank CPs (without GOX); iii: VEGF-loaded CP (neutral, without GOX); iv: VEGF-loaded CPs (acidic, without GOX). Scale bar is 50 μm.

Polysaccharide-based polymers are often employed as hemostatic materials. Here, we conducted *in vitro* coagulation test to investigate the hemostatic performance of the CPs. As shown in Fig. 4a, the CPs were mixed with sodium citrate containing blood, and saline was used to rinse the mixture at the corresponding time points. The formed fibrin clots that cannot be removed by saline would remain at the bottom of the well plate. For HAMA/Alg hydrogel, the presence of calcium ions can serve as a cofactor in several key stages of the coagulation cascade. Additionally, the polysaccharides ODex and QCS can promote platelet adhesion and aggregate red blood cells, thereby achieving hemostasis. The results indicated that both HAMA/Alg and CPs can rapidly induce blood clot formation, demonstrating excellent hemostatic property (Fig. 4b).

To evaluate the antibacterial performance, AMP-loaded CPs (with GOX) was set as one of the experimental groups (v). The other four groups included PBS group (i), HAMA/Alg hydrogel group (ii), the pure CPs group (without GOX) (iii), the AMP-loaded CPs group (without GOX) (iv). The accumulation of acidic products during bacterial growth and metabolism would cause pH decrease, further leading to the degradation of ODex/QCS (with GOX). Meanwhile, the presence of glucose would also trigger the pH reduction due to the GOX catalysis ability (Fig. 4c). As shown in Fig. 4d and Fig. S10, the groups with AMP addition exhibited superior antibacterial performance. Furthermore, comparing the results of group iv and group v, it was observed that in the presence of GOX, more bacteria were killed, highlighting the effectiveness of the proposed system in preventing bacterial infections, especially in high-glucose environments. Plate coating results also displayed similar trends (Fig. S11).

Biocompatibility is a crucial factor in the development of wound dressings. Here, the culture medium extracts of HAMA/Alg and ODex/QCS bulk hydrogel were used to culture 3T3 and HUVEC cells to assess the cytocompatibility of these materials. The fluorescent images of 3T3 cells in Fig. 4e and the CCK-8 tested cell viability results in Fig. S12 suggested that the hydrogels did not release toxic substances. The similar result also could be found in HUVECs (Fig. S13). The excellent biocompatibility of the material components made CPs suitable drug carriers. VEGF is a crucial promoter in angiogenesis, significantly contributing to the development of new blood vessels. To verify the angiogenic effect of VEGF, experiments were conducted using human umbilical vascular endothelial cells (HUVECs). As depicted in Fig. 4f, The HAMA/Alg and ODex/QCS hydrogel didn't promote vascular tube formation. However, after loading with VEGF, the effectiveness of cellular tube was significantly enhanced. Combined the hemostatic performance, antibacterial ability, biocompatibility and enhanced angiogenesis capacity, the CP system hold great potential for wound management.

**Fig. 5:**
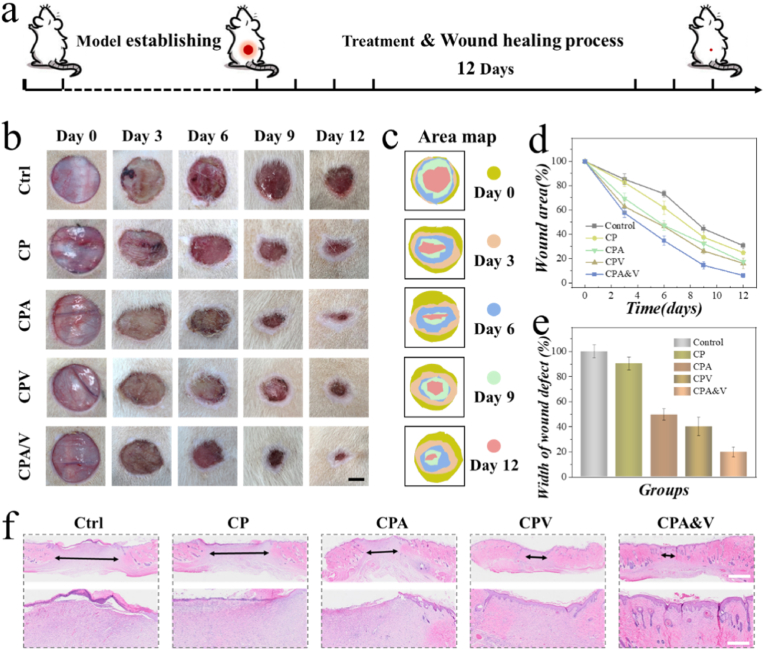
a) Schedule of animal experiment including model establishing, treatment, and healing process. b) Recorded wound images in different groups. Scale bar is 5 mm. c) Area map in different groups. d) Analysis of wound area in each group. e) Analysis of width of the wound defect in each group on Day 12. f) H&E staining of skin tissue taken from the rats on Day 12. Scale bars represent 2 mm (top), and 500 μm (bottom).

To further verify the therapeutic effect of drug-loaded CP *in vivo*, diabetic wound model was established in Sprague-Dawley (SD) rats. The scheme of the model establishment and treatment processes was shown in Fig. 5a. All rats were casually allotted to five groups. The group receiving PBS treatment served as the control. The remaining four groups were treated with blank CP (CP), AMP-loaded CP (CPA), VEGF-loaded CP (CPV), and AMP&VEGF-loaded CP (CPA&V), respectively. The changes in wound area for each group were recorded, as shown in Fig. 5b. It was evident that the CPA&V group exhibited the greatest extent of wound healing, suggesting better management effects compared to the other groups (Fig. 5c–e). Additionally, hematoxyline-eosin (H&E) staining was employed to further assess the healing quality of newly formed skin tissues. As shown in Fig. 5f, the skin samples from the drug-loaded groups, particularly the CPA&V group, demonstrated superior histological characteristics compared to that in the control and CP groups, including more complete re-epithelialization. These results indicated the promoting effect of the drugs in the healing process.

**Fig. 6:**
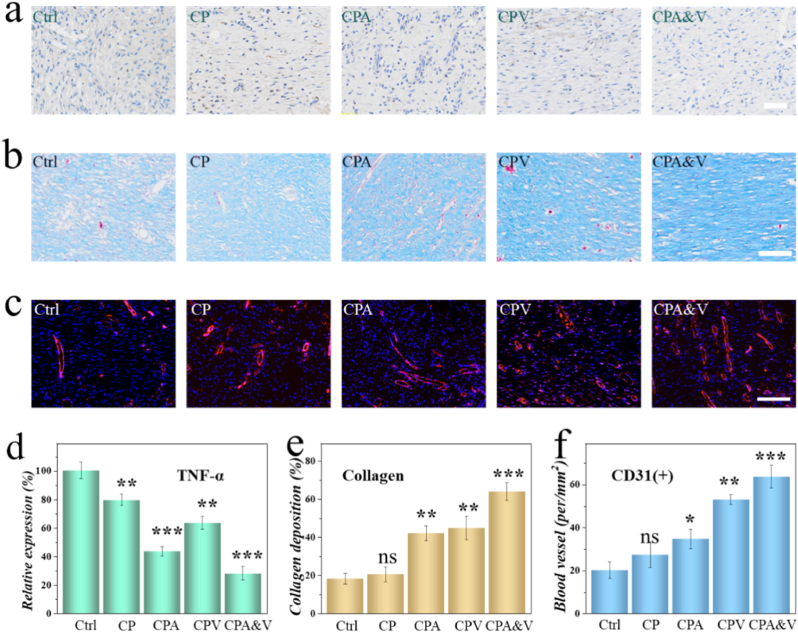
a) Immunohistochemical staining images of TNF-α across various groups. b) Masson staining images among each group. c) Images of immunofluorescence staining of CD31 in different groups. d-f) Analysis chart of relative expression of TNF-α (d), collagen deposition (e), and CD31 positive structures (f). Scale bars represent 100 μm in all.

Bacterial-infected wounds typically experience significant inflammatory cell infiltration. Therefore, immunohistochemical staining was performed to characterize the Tumor Necrosis Factor-alpha (TNF-α) expression in skin wounds. As shown in Fig. 6a and d, the CPA&V group exhibited the lowest TNF-α expression, notably lower than that in the control and CP groups. This indicated that the smart responsive drug-loaded CP effectively inhibited bacterial infection, thereby alleviating the inflammation. Additionally, considering that the collagen deposition degree reflects the wound healing outcome, Masson's trichrome staining was employed for evaluation (Fig. 6b and e). Both the control group and the CP group showed minimal collagen expression, while the CPA&V group displayed the most obvious collagen deposition, which is conductive to further wound management. Moreover, neovascularization can help transport nutrients and participate in metabolism, directly reflecting the wound healing rate. Fig. 6c illustrated the new blood vessels in the skin wounds of each group and the corresponding analysis was shown in Fig. 6f. In the absence of VEGF, the skin sample in control, CP and CPA group possessed lower vascular density. In contrast, the CPV group, especially the CPA&V group, showed more vascular positive expression, suggesting that the CPA&V system effectively promoted wound neovascularization. All these results demonstrated that the proposed drug-loaded CP possessed outstanding advantages in promoting wound management.

## 3 Conclusion

In summary, we have prepared a pH/glucose dual physiological responsive structural color particles for diabetic wound repair. Such particles were composed of a HAMA/Alg inverse opal scaffold and were further filled with AMP&VEGF&GOX-loaded ODex/QCS hydrogel. Within this system, the dual-network structure provides the scaffold with enhanced toughness and structural stability. Furthermore, the ODex/QCS hydrogel, with the integration of GOX, was characterized with pH/glucose dual responsiveness. When applied in wound, the particle system encounters the high-glucose environment of the wound. Then, GOX efficiently catalyzes glucose to generate acidic products, triggering the degradation of the ODex/QCS hydrogel. This process causes controllable release of the encapsulated AMP and VEGF. Importantly, this process also leads to changes in the particle's structural color because of the change of the refractive index, offering significant potential for wound monitoring. It has been demonstrated that such particles greatly promoted the healing progress of diabetic wound *in vivo*. It is convinced that such dual physiological responsive structural color hydrogel particles will find great potential for wound management.

## 4 Materials and methods

**Materials.** Methacrylic anhydride, photoinitiator 1173 (HMPP), and sodium alginate were obtained from Sigma-Aldrich (USA). Quaternized chitosan, hyaluronic acid, AMP (eumenitin), and CCK-8 reagents were acquired from Beyotime Biotechnolog CO., LTD (China). Other reagents were all achieved from Macklin reagent (China).

**Preparation and characterization of ODex.** 5g of Dextran was dissolved in 75 mL water. Then, sodium periodate (NaIO_4_) was applied to oxidize dextran. In brief, 1.32g of NaIO_4_ was dissolved in 20 mL water. To adequately react with dextran, the NaIO_4_ solution was added into reaction system drop by drop. After 24h incubation in the dark, the reaction was stopped by adding plenty of ethanol. Then, the reaction solution was transferred to a suitable dialysis bag and dialyzed in pure water for 72 h. The ODex was prepared through a freeze-drying process and further characterized.

**Fabrication and characterization of HAMA/Alg inverse opal scaffold.** The inverse opal particles were achieved by reversely replicating silica photonic crystal template. The silica photonic crystal template and HAMA polymer were synthesized according to previous work [23,29]. To fabricate the pregel solution, 1g of HAMA and 0.1g Alg were dissolved in 10 mL water. The photoinitiator HMPP concentration adjusted to 1 %. The dried template was immersed in the pregel solution. The system was first photocured by an ultraviolet (UV) light source and then put into calcium chloride solution for 1 h. Then, the hybrid particles were isolated from the system and immersed in hydrofluoric acid solution. After that, HAMA/Alg inverse opal scaffold was ultimately obtained. SEM was utilized to study the micro/nano structure of particles, and resin was used for shooting.

**Fabrication and characterization of ODex/QCS hydrogel.** ODex and QCS were respectively dissolved in water to obtain the solution. Then, the ODex solution and QCS solution were mixed. The final concentration of ODex and QCS in the mixed solution were 4 % and 2 %, respectively. Under neutral conditions, the two polymers could react to form dynamic hydrogel. Then, the gelation progress and the chemical state of the dynamic hydrogel was analyzed through rheological measurement and X-ray photoelectron spectroscopy (XPS).

**Fabrication of composite hydrogel particles.** To better refill the nanovoids of the particles, the inverse opal scaffold was first undergone dehydration at 37 °C for 2 h. Then, such scaffold was immersed in ODex and QCS mixed solution with GOX (0.1 mg/mL) doped. Then, the mixed system was centrifuged for 20 min. After gelation, the CPs were separated from the system for further application.

**Biocompatibility test.** HAMA/Alg hydrogel and ODex/QCS hydrogel (without GOX) were first immersed in culture medium for 24h, respectively. Then, 8 × 10^3^ 3T3 cells were seeded into the 48-well plate. Various material leachates were introduced into these wells, and cell viability was detected by using CCK-8 kit on Day 1, Day 2, and Day3. Besides, the 3T3 cells in corresponding groups were stained through calcein to evaluate the cell viability. Meanwhile, we also use HUVECs to test the biocompatibility, and cell viability was measured on Day 3.

**ODex/QCS hydrogel *in vitro* degradation test.** The ODex/QCS hydrogel (with GOX) was immersed in PBS (pH = 7.4 or 5.5). 0.5 mL of hydrogel was placed in a centrifuge tube, followed by adding of the PBS buffer with different pH values. The centrifuge tubes were kept at 37 °C and shaken at a speed of 100 rpm/min. At each time point, the hydrogel was rinsed with deionized water and weighed. The remaining weight of the hydrogel was recorded by comparing its current weight to its initial weight. To evaluate the degradability of ODex/QCS (with GOX) in the presence and absence of glucose, PBS glucose solution (5 mM) with an initial pH of 7.4 was used for the experiment, and the subsequent procedure were the same as described above.

***In vitro* coagulation test.** Whole blood was sampled and stored in anticoagulation tube. When the blood was removed from the anticoagulant tube, a 0.1 M calcium chloride solution was mixed and stirred for 10 s. HAMA/Alg hydrogel and CPs (without GOX) were placed at the 96-well plate (5 mg). Subsequently, 50 μL of the mixed blood was introduced into every well. At designated time points, each well was rinsed with saline to remove the unclotted substances.

***In vitro* drug release.** FITC-BSA (EX: 490 nm; EM: 525 nm) and Rhodamine B-labeled AMP (EX: 555 nm; EM: 605 nm) were applied to explore drug release behavior of CPs. The fluorescent images of CPs were captured through confocal microscopy. The drug-loaded CPs (with GOX) were placed in PBS solution (pH = 5.5 or 7.4), respectively. To simulate *in vivo* conditions, the drug release behavior was monitored at 37 °C with shaking (100 rpm/min). At predesigned time points, 200 μL of the solution was sampled and replaced with fresh PBS. The fluorescence intensity of the released drug was detected using a microplate reader.

**Tube formation test.** 3 × 10^4^ HUVECs were planted into a 48-well plate pre-coated with growth factor-reduced Matrigel. Then, these wells were divided into four groups, including control group, blank CPs (without GOX), VEGF-loaded CPs (neutral, without GOX), and VEGF-loaded CPs (acidic, without GOX). Notably, to avoid the influence of pH, the cells in the experimental group were cultured with fresh medium mixed with the leaching solution. In the VEGF-loaded CPs (acidic, without GOX) group, the pH value of leaching solution is 5.5. After culturing for 6h, the treated cells were stained and captured with an inverted fluorescence microscope.

**Antibacterial test.** To evaluate the antibacterial efficacy of the particle system, *E. coli* and *S. aureus* were taken as representative bacteria strains. First, bacteria in the logarithmic growth phase were centrifuged and resuspended in PBS containing glucose. The resuspended solution was then treated with four different agents at 37 °C including the PBS group, HAMA/Alg hydrogel group, the pure CPs group (without GOX), the AMP-loaded CPs group (without GOX), and the AMP-loaded CPs group (with GOX), respectively. After culturing, a live/dead kit was performed to assess viability of these bacteria. Then, fluorescence pictures were captured using a fluorescence microscope.

***In vivo* animal test.** Animal tests were approved by the Animal Ethical Committee of the Wenzhou Institute, University of Chinese Academy of Science (WIUCA23041304). The 5- to 6-week-old Sprague-Dawley rats were acquired. To establish diabetes, the rats were administered with streptozotocin (1 % (w/v), 65 mg/kg). When each rat's blood glucose level reached the desired standard and stabilized, a full-thickness circular wound (about 1.5 cm) was created on rat back, followed by the addition of bacterial suspension. Subsequently, these rats were erratically assigned into five groups and treated with different intervention therapies, including PBS-rinsing group (Control), pure GOX-doped CPs group (CP), AMP&GOX-loaded CPs group (CPA), VEGF&GOX-loaded CPs group (CPV), and AMP&VEGF&GOX-loaded CPs group (CPA&V). The wounds were observed every day and photographed on Day 0, 3, 6, 9, and 12. On the last day, all rats were euthanized, and their wound tissues were collected and subjected to further analysis.

## CRediT authorship contribution statement

**Li Wang:** Writing – original draft, Formal analysis, Data curation. **Lu Fan:** Writing – review & editing, Methodology, Data curation. **Anne M. Filppula:** Writing – review & editing, Methodology. **Yu Wang:** Supervision, Writing – review & editing. **Feika Bian:** Writing – review & editing, Supervision, Conceptualization. **Luoran Shang:** Writing – review & editing, Supervision, Funding acquisition, Conceptualization. **Hongbo Zhang:** Writing – review & editing, Supervision, Funding acquisition, Conceptualization.

## Ethics approval and consent to participate

Animal tests were approved by the Animal Ethical Committee of the Wenzhou Institute, University of Chinese Academy of Science (WIUCA23041304).

## Declaration of competing interest

Hongbo Zhang is an editorial board member for Bioactive Materials and was not involved in the editorial review or the decision to publish this article. All authors declare that there are no competing interests.
