# Supplementary material for: Dual physiological responsive structural color hydrogel particles for wound repair
Source: Bioact Mater. 2025 Jan 7;46:494–502. doi: 10.1016/j.bioactmat.2025.01.002 (PMC11760816; doi:10.1016/j.bioactmat.2025.01.002)
Supplement: Multimedia component 1 [file mmc1.docx]

**Dual physiological responsive structural color hydrogel particles for wound repair**


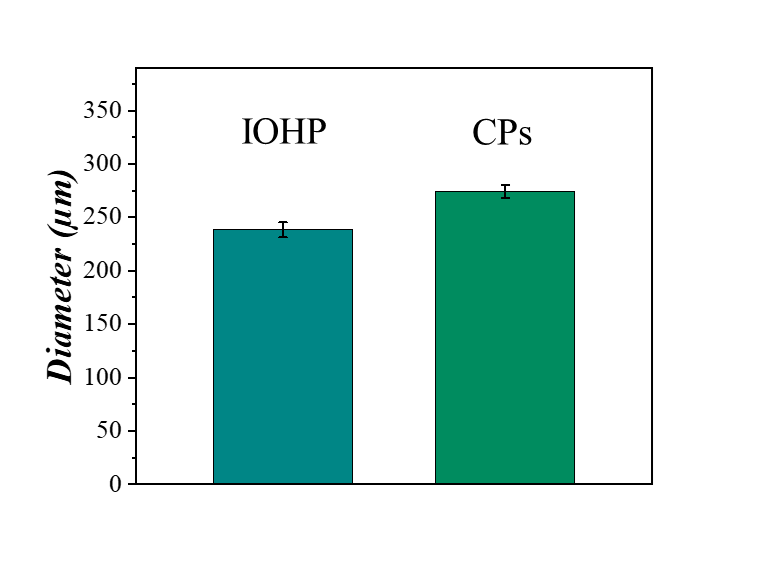


**Figure S1.** Particle size analysis of IOHP and CPs.


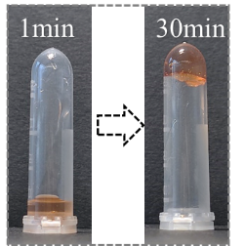


**Figure S2.** a) Images of ODex/QCS hydrogel at 1min and 30min after gelation.


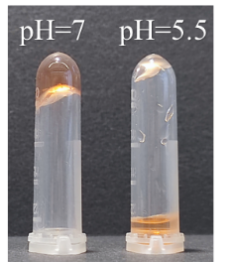


**Figure S3.** Images of ODex/QCS system at different pH value.


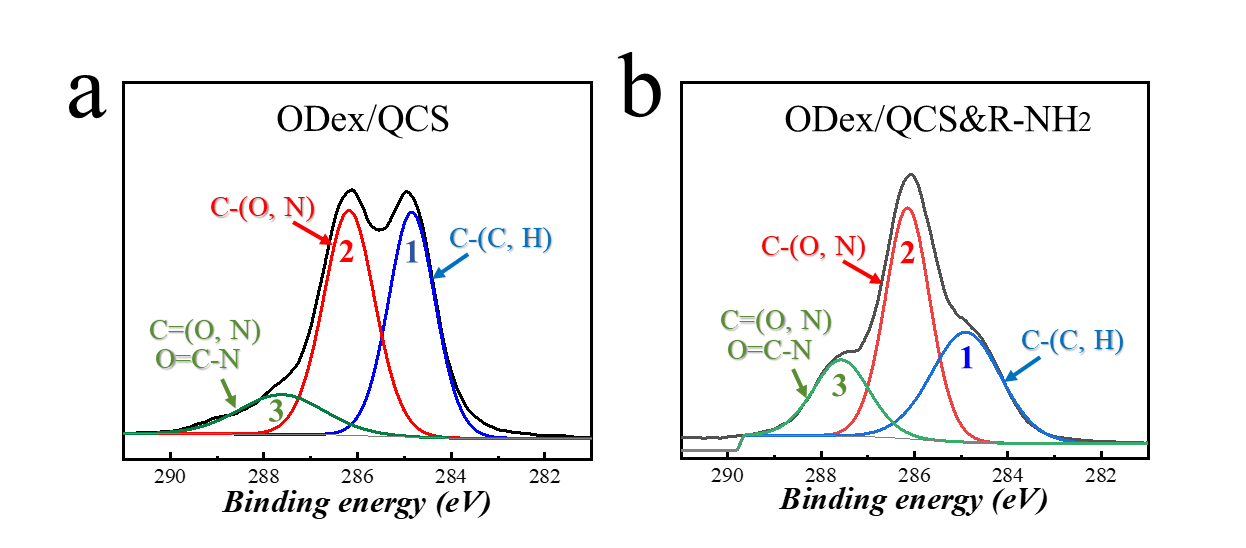


**Figure S4.** XPS C 1s spectra of ODex/QCS and ODex/QCS&R-NH_2_.


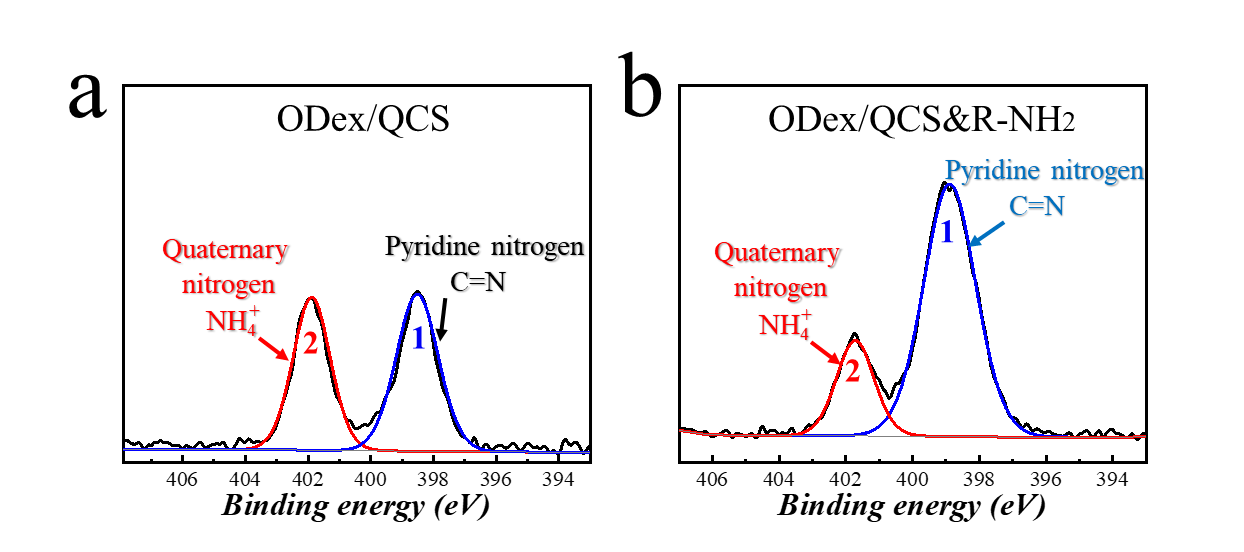


**Figure S5.** XPS N 1s spectra of ODex/QCS and ODex/QCS&R-NH_2_.


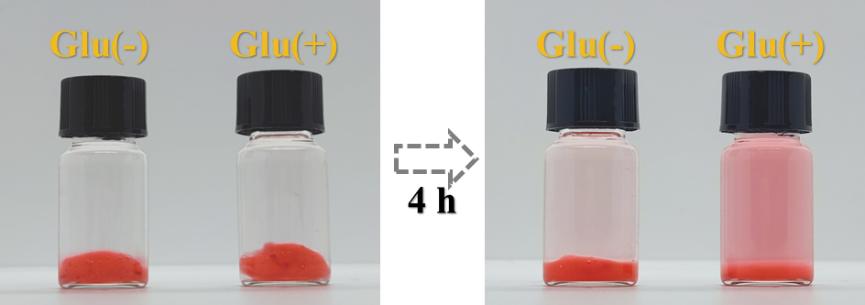


**Figure S6.** ODex/QCS (with GOX) in a PBS buffer solution with or without glucose (5 mM).

**Figure S7.** Analysis of remaining weight of GOX-doped ODex/QCS hydrogel in PBS buffer with or without glucose (5 mM).


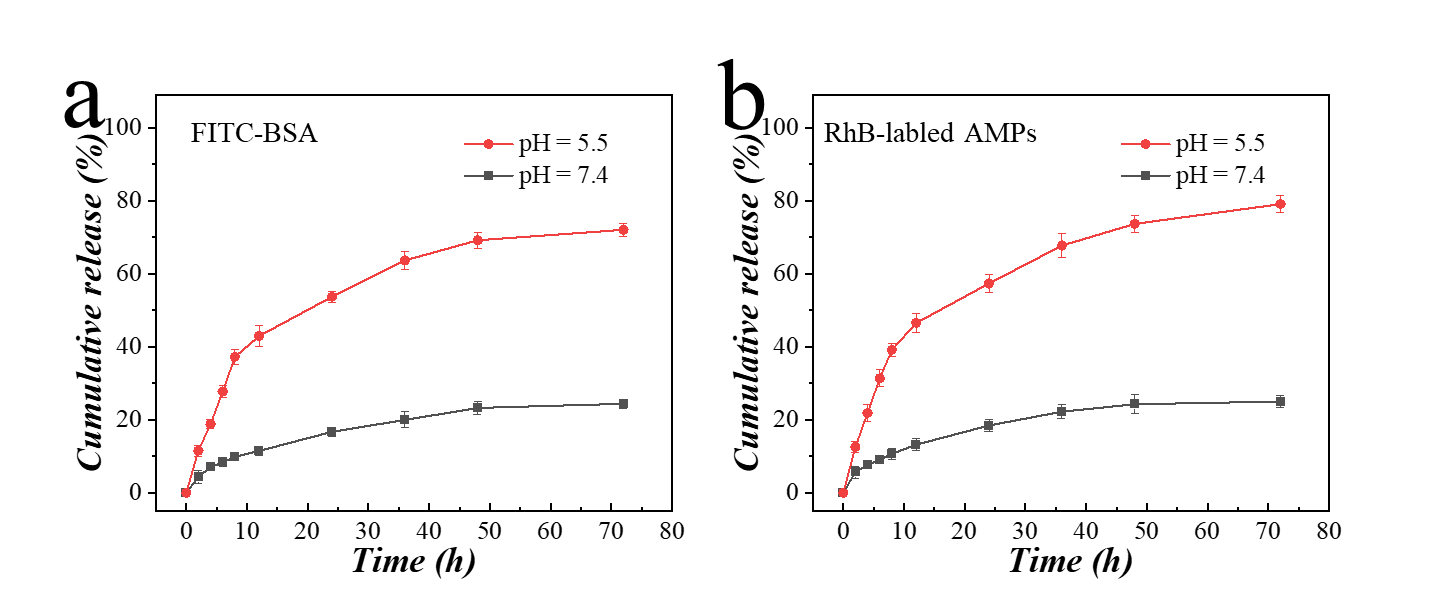


**Figure S8.** Release behaviours of FITC-BSA and Rhodamine B-labelled AMP in GOX-doped ODex/QCS hydrogel in PBS with different pH value.


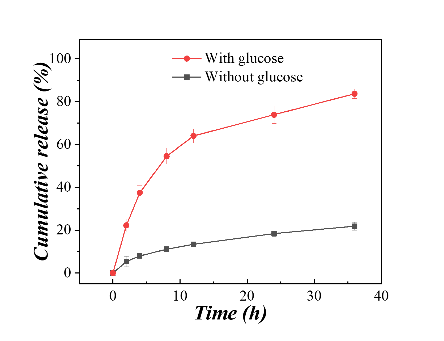


**Figure S9.** Release behaviours of Rhodamine B-labelled AMP in GOX-doped ODex/QCS hydrogel in PBS with or without glucose (5 mM).


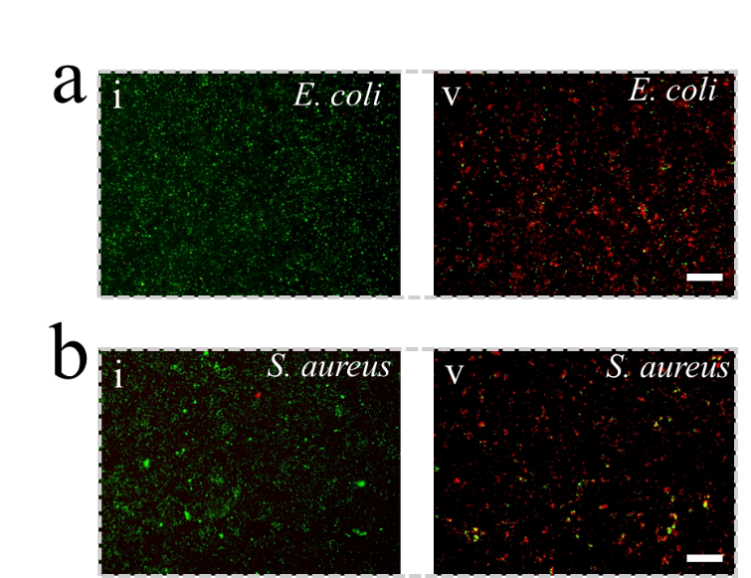


**Figure S10.** Live/Dead florescent staining of *E. coli* and *S. aureus* with long-term treatment: i) PBS group; v) the AMP-loaded CPs group (with GOX). Scale bars are 100 µm.


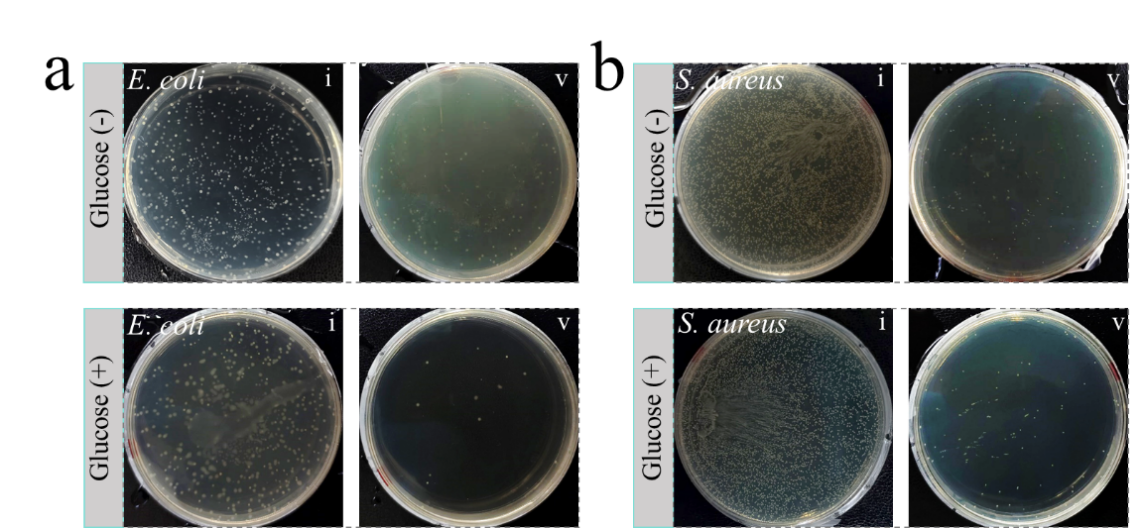


**Figure S11.** Plate coating results of *E. coli* and *S. aureus* under different treatment: i) PBS group; v) the AMP-loaded CPs group (with GOX). The group labeled glucose (+) were supplemented with glucose.

**Figure S12.** Analysis of OD value in different groups on Day 1, Day 2, and Day 3.


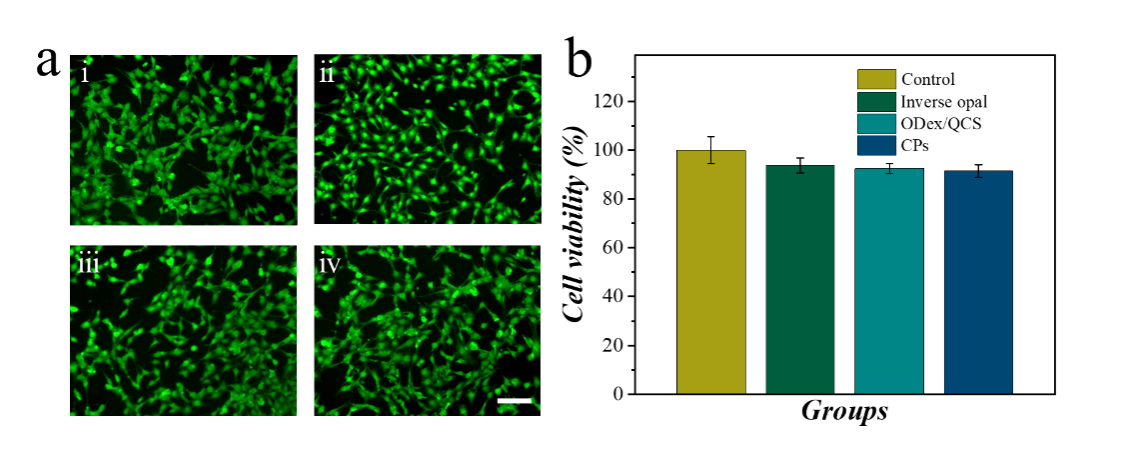


**Figure S13.** Florescent images of HUVECs and corresponding cell viability in different groups. i: control group; ii: HAMA/Alg hydrogel group; iii: ODex/QCS hydrogel group; iv: HAMA/Alg and ODex/QCS composite material group. Scale bar is 50 µm.
